# Missed opportunities: racial and neighborhood socioeconomic disparities in emergency colorectal cancer diagnosis and surgery

**DOI:** 10.1186/1471-2407-14-927

**Published:** 2014-12-09

**Authors:** Sandi L Pruitt, Nicholas O Davidson, Samir Gupta, Yan Yan, Mario Schootman

**Affiliations:** Department of Clinical Sciences, University of Texas Southwestern Medical Center, 5323 Harry Hines Blvd E1, 410D Dallas, TX USA; Harold C. Simmons Comprehensive Cancer Center, Dallas, TX USA; Department of Medicine, Division of Gastroenterology, Washington University School of Medicine, Saint Louis, MO USA; Alvin J. Siteman Cancer Center at Barnes-Jewish Hospital, Washington University School of Medicine, Saint Louis, MO USA; Department of Internal Medicine, Division of Gastroenterology, Moores Cancer Center, University of California, San Diego, CA USA; Department of Veterans Affairs, San Diego Healthcare System, San Diego, CA USA; Department of Surgery, Division of Public Health Sciences, Washington University School of Medicine, St Louis, MO USA; College for Public Health and Social Justice, Saint Louis University, St. Louis, MO USA

**Keywords:** Colorectal cancer, Emergency outcomes, Disparities, Race, Socioeconomic status, SEER-Medicare

## Abstract

**Background:**

Disparities by race and neighborhood socioeconomic status exist for many colorectal cancer (CRC) outcomes, including screening use and mortality. We used population-based data to determine if disparities also exist for emergency CRC diagnosis and surgery.

**Methods:**

We examined two emergency CRC outcomes using 1992–2005 population-based U.S. SEER-Medicare data. Among CRC patients aged ≥66 years, we examined racial (African American vs. white) and neighborhood poverty disparities in two emergency outcomes defined as: 1) newly diagnosed CRC or 2) CRC surgery associated with: obstruction, perforation, or emergency inpatient admission. Multilevel logistic regression (patients nested in census tracts) analyses adjusted for sociodemographic, tumor, and clinical covariates.

**Results:**

Of 83,330 CRC patients, 29.1% were diagnosed emergently. Of 55,046 undergoing surgery, 26.0% had emergency surgery. For both outcomes, race and neighborhood poverty disparities were evident. A significant race by poverty interaction (p < .001) was noted: poverty rate was associated with both outcomes among African Americans, but not whites. Compared to whites in low poverty (<10%) neighborhoods, African Americans in high poverty (≥20%) neighborhoods had increased odds of emergency diagnosis (AOR: 1.50, 95% CI: 1.38-1.63) and surgery (AOR: 1.63, 95% CI: 1.47-1.81).

**Conclusions:**

Emergency CRC outcomes are associated with high poverty residence among African Americans in this population-based study, potentially contributing to observed disparities in CRC morbidity and mortality. Targeted efforts to increase CRC screening among African Americans living in high poverty neighborhoods could reduce preventable disparities.

## Background

Colorectal cancer (CRC) screening results in dramatically improved survival [1, 2] and can prevent late-stage disease and associated serious and emergent complications including obstruction, perforation, hemorrhage, and peritonitis. These complications, when presenting acutely, are considered emergencies—posing immediate risks to life or long-term impairment—and require immediate intervention including surgical management and intensive care unit stays. Of those diagnosed emergently and receiving curative resection, both short- and long-term outcomes, including mortality and medical and surgical complications, are significantly worse than those receiving elective curative resection [3–5]. For example, a U.S. National Inpatient Sample study demonstrated that emergency resection was associated with a 3-fold increase of in-hospital mortality, longer hospital stays, and greater hospital costs [6]. Emergency resection is also associated with suboptimal long-term outcomes. A U.S. Surveillance Epidemiology and End Results (SEER)-Medicare study of 30,685 stage I-III colon cancer patients found that non-elective resection was associated with significantly worse five-year disease-specific survival (Hazard Ratio: 1.30, p < .001), even after adjustment for pre-, peri-, and post-operative covariates [7].

CRC emergencies may disproportionately affect African Americans and low socioeconomic status (SES) populations. For example, in a U.S. population-based study of 127,975 CRC patients undergoing resection in 2002, African Americans were more likely to have emergency resection [6]. However, in the same study, median income of patients’ zip code was not associated with emergency resection [6]. Studies from England and Canada, however, demonstrate that CRC patients living in low SES neighborhoods are more likely to be diagnosed emergently[4, 8, 9]. African American and disadvantaged, low-SES populations are less likely to have obtained and maintained cancer screening than their counterparts and partly as a result, experience a disproportionate cancer burden across numerous CRC outcomes [10–12]. For example, compared to whites, African American men and women have a 22-23% higher CRC incidence rate and a 46-53% higher CRC mortality rate, respectively [13]. In a study comparing those living in counties where <10% population lived in poverty, both African Americans (OR: 1.13; 95% CI: 1.03-1.25) and whites (OR: 1.10; 95% CI: 1.06-1.15) living in counties with ≥20% poverty had higher odds of distant stage CRC [14].

The overwhelming majority of studies on emergency diagnosis or surgery of CRC originate from Europe or Canada, include small samples, and are not population-based. Thus, the U.S. experience, particularly race and SES disparities, has not been adequately studied. Further, while there is documented geographic variation in CRC outcomes such as screening and mortality, [15–20] the extent of neighborhood variation in CRC emergencies is unknown. Neighborhood variation indicates that shared neighborhood-level characteristics, in addition to individual characteristics, are associated with emergencies, which could suggest a role for geographically-targeted prevention strategies.

To our knowledge, there is only one population-based U.S. study on emergency CRC *diagnosis,* [21] and none that concurrently examine both emergency diagnosis and surgery. Many patients diagnosed emergently have advanced disease, multimorbidity, low functional status, or poor prognosis, which could postpone or preclude curative resection, particularly African Americans and those of low SES who often present with more advanced disease [14]. Thus, there is a paucity of information about the distribution and correlates of emergency diagnosis (with or without emergency curative resection) in the U.S.

Here we describe 1) variation across neighborhoods and 2) race and neighborhood SES disparities in two outcomes, emergency CRC diagnosis and surgery, among U.S. CRC patients aged ≥66 years. Given lower rates of screening and worse CRC morbidity and mortality among African Americans [6, 10, 12, 13] and patients in low SES neighborhoods [4, 8, 9, 14], we hypothesized these populations would have higher rates of both emergency outcomes.

## Methods

### Data sources

Data were obtained from an existing linkage of the 1992–2005 National Cancer Institute’s (NCI) SEER program data with 1991–2006 Medicare claims files from the Centers for Medicare and Medicaid. Data access was granted by the NCI. As detailed elsewhere, [22] linked SEER-Medicare data provide a rich source of information on Medicare patients included in SEER, a nationally representative collection of population-based cancer registries. Ninety-four percent of cancer patients reported to SEER aged 65 years or older have been successfully linked with Medicare data [22]. Data for this study were available from 12 registries representing approximately 14% of the U.S. population, [23] including states (Connecticut, Hawaii, Iowa, New Mexico, and Utah), metropolitan areas (Atlanta, Detroit, Los Angeles, San Francisco-Oakland, San Jose-Monterey, and Seattle), and rural Georgia. This study was reviewed by the Institutional Review Board at Washington University and determined to be exempt from IRB oversight.

### Study population

The study population included male and female patients aged ≥66 with a diagnosis of a first primary invasive colorectal cancer occurring from 1992 through 2005. We included only those aged ≥66 to allow for one-year of complete claims data pre-diagnosis to determine comorbidity. All patients had full coverage by both Medicare Parts A and B from one year pre-diagnosis until 120 days past primary surgery or until initiation of adjuvant chemotherapy, whichever came first. We excluded patients with appendix cancer, only autopsy or death certificate records, and members of HMOs. We excluded patients with a diagnosis of Crohn’s disease, ulcerative colitis, inflammatory bowel disease, or diverticulitis (ICD-9 556.X, 555.X, 562.01, 562.03, 562.11, 562.13) in the year prior to diagnosis. Finally, we also excluded patients (n = 5,529) missing year 2000 census tract in the SEER-Medicare data.

For the emergency surgery analysis, we included only patients with CRC surgeries (identified using ICD9 procedure codes described below) and excluded patients with abdominal or pelvic postoperative adhesions (ICD-9 560.81, 568.0, 614.6) in the pre-surgery year due to a high likelihood of emergency surgery for these conditions. We further excluded patients with neoadjuvant chemotherapy or radiation occurring in the year until 7 days before surgery as an emergency in this situation could merely reflect complications of a preexisting cancer [24].

### Study variables

#### Emergency diagnosis and surgery

We defined emergency diagnosis and surgery as yes/no variables by the presence of any of 3 emergency Medicare claims indicators based on ICD9 codes and an inpatient admission type indicator, including: 1) obstruction (560.89, 560.9); 2) perforation (569.83); or 3) emergency inpatient admission (admtype = 1; patient required immediate medical intervention as a result of severe, life threatening, or potentially disabling conditions). These indicators were identified from previous literature [6, 7, 9, 24–26] and were selected because they are highly correlated with CRC emergencies; emergency surgeries are commonly a result of obstruction and/or perforation [7]. Further, chosen indicators are unlikely to identify nonemergent symptoms or unrelated conditions (e.g. rectal bleeding is relatively nonspecific and could indicate minor bleeding or hemorrhoids). For inclusion as an emergency outcome, we specified that dates associated with the emergency indicators fall within 1) SEER month and year of initial CRC diagnosis (because day of diagnosis is not available in SEER) (emergency diagnosis); or 2) within ±3 days of the first of any CRC surgery (e.g. resections, pelvic exenteration, colostomy, [not including colostomy reversals]) (emergency surgery).

#### Race and socioeconomic (SES) status

We compared non-Hispanic African Americans to non-Hispanic whites. We defined SES as the percent of population living in poverty in the patient’s residential census tract at time of CRC diagnosis. We selected this indicator because census tract-level poverty rate is a robust indicator of SES, is associated with a variety of health outcomes in diverse populations and across time, and has relevance for policymakers [27, 28]. We used 2000 U.S. Census data using the following commonly used categories: <9.9%, 10–19.9%, and ≥20% [29, 30].

#### Covariates

Multiple covariates associated with emergency CRC outcomes in earlier studies [6, 7, 9, 21] were examined. Covariates obtained from SEER data included: year of diagnosis, age (66–69, 70–74, 75–79, 80–84, ≥85), sex, SEER historic stage (localized, regional, distant), tumor location (right [cecum: 18.0, ascending colon: 18.2, hepatic flexure of colon: 18.3, and transverse colon: 18.4] vs. left [splenic flexure of colon: 18.5, descending colon: 18.6, sigmoid colon: 18.7, rectosigmoid: 19.9, rectum: 20.9]), histology (mucinous adenocarcinoma/signet ring cell, other adenocarcinoma, other, unknown), and tumor grade (low [well/moderately differentiated] or high [poorly differentiated/ undifferentiated/anaplastic] or unknown).

Covariates obtained from Medicare claims included: prior hospitalizations, comorbidity, preventable hospitalizations, number of endoscopies, and Medicaid and Medicare (“dual eligibility”). Prior hospitalizations occurring between 12–1 months prior to diagnosis were measured as yes/no. To measure comorbidity, we searched inpatient or carrier claims for multiple chronic conditions (e.g. myocardial infarction, diabetes, dementia, or AIDS) occurring between 12–1 months pre-diagnosis using Charlson comorbidity index-Klabunde adaptation [31, 32]. We further classified comorbidity as none, one, or two or more following common practice. Preventable hospitalizations identify poor ambulatory health care outcomes and can represent a breakdown in access to or processes of primary care. Following methods described elsewhere, [33] we searched one-year pre-diagnosis of inpatient claims for potentially preventable hospitalizations, including asthma, diabetes, hypertension, pneumonia, and compared those with one or more preventable hospitalizations to those with none. Because claims provide accurate information on endoscopy procedures but are less reliable for stool blood testing and cannot distinguish screening from diagnostic tests, [34] we measured total number of endoscopies (colonoscopies and sigmoidoscopies) in the pre-diagnosis year, but were not able to control for complete CRC screening history starting at age 50. Dual-eligibility was defined as Medicaid eligibility for at least 1 month during the pre-diagnosis year. Census-tract urban/rural status (metropolitan, micropolitan, or rural) was measured using 2000 Census Rural Urban Continuum Area codes [35].

### Statistical analysis

We describe sample characteristics and frequency of emergency diagnosis and emergency surgery using descriptive and chi-square statistics. We examine associations of race and neighborhood poverty rate, including a race by poverty interaction term, with both outcomes. We present 6 models: 1) empty model; 2) unadjusted race model; 3) unadjusted poverty model; 4) unadjusted race and poverty model; 5) adjusted race and poverty main effects model; and 6) adjusted race and poverty interaction model. Empty models include no predictor variables, but include a hierarchical structure, and are fit for the purpose of quantifying variation (random effects) at the census tract level. We multiplied race and poverty rate variables to determine if an interaction was present on a synergistic scale and retained the main effects of race and poverty in the model. Covariates were retained if they were associated with outcomes in bivariate analyses (p < .05). All models were fit using multilevel logistic regression to account for nesting of patients within residential census tracts, using SAS PROC GLIMMIX (SAS Institute Inc, Cary, NC).

To facilitate interpretation of census-tract (“neighborhood”) level random effects, we present variance and standard error and Median Odds Ratio (MOR). The MOR quantifies unexplained cluster heterogeneity [36, 37] on a scale directly comparable to odds ratios associated with other model variables [38]. MOR is based on the random effects variance component (V) from the regression model: . It is interpreted as the median value of the ratio of predicted odds of the outcome for two patients with equivalent covariates randomly chosen from two different neighborhoods. MOR is always ≥1; 1.0 indicates no variation between neighborhoods and larger values indicate greater geographic variation.

In a sensitivity analysis, we disaggregated each emergency outcome into 2 component variables (emergencies defined as obstruction and/or perforation [because only 1.1% and 1.2% of all diagnoses and surgeries, respectively, were associated with perforations] and emergency admission type [admtype = 1]) and re-fit all multilevel logistic regression models separately for each outcome.

## Results

Of all 83,330 eligible patients, 29.1% had an emergency diagnosis. Of all patients undergoing surgery (n = 55,046), 26.0% had an emergency surgery. Table 1 presents sample characteristics by presence of emergency diagnosis and surgery status. All but one of the sample characteristics differed (p < .001) by emergency status and were included in the adjusted models. Occurrence of emergency diagnosis and emergency surgery did not significantly change over time (p > .05); therefore year of diagnosis was not included in the adjusted models. Of patients diagnosed emergently and undergoing surgery, 84.0% had emergency surgery. Of patients undergoing emergency surgery, nearly all (94.0%) were also classified as having an emergency diagnosis (data not shown).

**Table 1:** **Sample characteristics of colorectal cancer patients, by emergency diagnosis and emergency surgery status**

	Diagnosis (n = 83,330) ^a^					Surgery (n = 55,046) ^a^				
Characteristic	No emergency diagnosis (n = 59,082)		Emergency diagnosis (n = 24,248)		p (Chi ^2^ )	No emergency surgery (n = 40,740)		Emergency surgery (n = 14,306)		p (Chi ^2^ )
	n	%	n	%		n	%	n	%	
Race										
White	54527	71.90	21313	28.10	<.0001	37957	75.01	12643	24.99	<.0001
African American	4555	60.81	2935	39.19		2783	62.60	1663	37.40	
Neighborhood poverty rate										
<10%	35999	71.72	14198	28.28	<.0001	25254	74.81	8505	25.19	<.0001
10-19.9%	14902	71.98	5801	28.02		10170	75.09	3374	24.91	
≥20%	8181	65.82	4249	34.18		5316	68.66	2427	31.34	
Sex										
Male	27154	73.36	9860	26.64	<.0001	18215	75.71	5843	24.29	<.0001
Female	31928	68.94	14388	31.06		22525	72.69	8463	27.31	
Age (mean [SD])	77.2	7.1	79.6	7.6	<.0001	76.8	6.8	78.9	7.4	<.0001
Year										
1992-1996	24621	70.68	10214	29.32	0.4126	9311	74.05	3263	25.95	0.9327
1997-2001	13092	71.21	5292	28.79		14847	74.08	5195	25.92	
2002-2005	21369	70.97	8742	29.03		16582	73.93	5848	26.07	
Medicaid enrollee										
No	52170	72.54	19753	27.46	<.0001	36395	75.51	11802	24.49	<.0001
Yes	6912	60.59	4495	39.41		4345	63.44	2504	36.56	
Rural urban commuting area										
Metropolitan	46093	69.38	20346	30.62	<.0001	31657	72.45	12037	27.55	<.0001
Micropolitan	5246	76.23	1636	23.77		3595	78.91	961	21.09	
Rural	7743	77.36	2266	22.64		5488	80.75	1308	19.25	
Stage										
Local	25470	78.60	6936	21.40	<.0001	17622	79.48	4549	20.52	<.0001
Regional	20160	69.12	9006	30.88		16936	72.69	6363	27.31	
Distant	9840	60.94	6307	39.06		5436	64.66	2971	35.34	
Tumor location										
Left	31557	73.93	11127	26.07	<.0001	20204	75.47	6568	24.53	<.0001
Right	25162	68.09	11790	31.91		19953	73.04	7366	26.96	
Tumor Grade										
Low	40222	72.40	15337	27.60	<.0001	30066	75.09	9976	24.91	<.0001
High	10551	68.11	4941	31.89		8134	72.08	3150	27.92	
Histology										
Other adenocarcinoma	49541	71.73	19526	28.27	<.0001	35208	74.46	12078	25.54	<.0001
Mucinous adenocarcinoma/signet ring cell	6252	68.95	2816	31.05		4920	73.01	1819	26.99	
Other	2365	64.09	1325	35.91		544	61.40	342	38.60	
Comorbidity										
0	34535	73.76	12284	26.24	<.0001	24359	76.19	7613	23.81	<.0001
1	14000	69.73	6077	30.27		9889	73.86	3500	26.14	
≥2	9628	62.06	5887	37.94		6492	67.03	3193	32.97	
Preventable hospitalization										
No	55900	72.62	21077	27.38	<.0001	38522	75.20	12704	24.80	<.0001
Yes	3182	50.09	3171	49.91		2218	58.06	1602	41.94	
Prior hospitalization					<.0001					
No	38866	82.62	8178	17.38		23910	82.93	4920	17.07	
Yes	20216	55.71	16070	44.29		16830	64.20	9386	35.80	
Endoscopies/prior year										
None	32028	67.93	15118	32.07	<.0001	19435	69.11	8685	30.89	<.0001
1	16806	75.27	5521	24.73		13345	80.29	3277	19.71	
≥2	10248	73.96	3609	26.04		7960	77.25	2344	22.75	

^a^Numbers may not add up to total due to missing data.

### Emergency diagnosis

Table 2 presents results from unadjusted and adjusted multilevel analyses. Patients in the diagnosis sample (n = 83,330) were nested within 14,191 census tracts (range: 1–87 patients per tract). In all unadjusted and adjusted main effects models, emergency diagnosis was more likely for African Americans compared to whites, and those living in the highest poverty census tracts, compared to those in the lowest poverty census tracts. In the adjusted main effects model (Model 5), African Americans (vs. whites) (OR 1.28 [95% CI: 1.20-1.37]) and those living in census tracts with the highest poverty rates (≥20% vs. <10%) (OR 1.11 [95% CI: 1.04-1.18]) were more likely to have emergency diagnosis.

**Table 2:** **The unadjusted and adjusted main effects between race and neighborhood poverty rate on emergency diagnosis and emergency surgery and neighborhood random effects**

Emergency diagnosis (n = 83,330 patients, 14,191 census tracts)										
Characteristic	Model 1 empty ^a^ model Model		Model 2 unadjusted race model		Model 3 unadjusted poverty model		Model 4 unadjusted race and poverty model		Model 5 adjusted ^b^ race and poverty main effects model	
	Odds ratios and 95% confidence intervals									
Race										
White	-		1		-		1		1	
African American	-		**1.58**	**1.50-1.67**	-		**1.48**	**1.40-1.57**	**1.28 (1.20-1.37)**	
Neighborhood poverty rate										
<10%	-		-		1		1		1	
10-19.9%	-		-		1.01	0.97-1.05	0.98	0.94-1.02	0.98 (1.04-1.18)	
≥20%	-				**1.32**	**1.26-1.39**	**1.17**	**1.11-1.23**	**1.11 (1.04-1.18)**	
	Neighborhood random effects									
Variance (se)	.4317 (.0114)		.4073 (.0110)		.4225 (.0112)		.4061 (.0110)		.4887 (.0157)	
Median odds ratio	1.87		1.84		1.86		1.84		1.95	
**Emergency surgery (n = 55,046 patients, 12,886 census tracts)**										
**Characteristic**	**Model 1** ^**a**^		**Model 2**		**Model 3**		**Model 4**		**Model 5** ^**b**^	
	Odds ratios and 95% confidence intervals									
Race										
White	-		1		-		1		1	
African American	-		**1.74**	**1.63-1.87**	-		**1.63**	**1.51-1.76**	**1.33**	**1.23-1.44**
Neighborhood poverty rate										
<10%	-		-		1		1		1	
10-19.9%	-		-		1.01	0.96-1.06	0.97	0.92-1.02	0.98	0.92-1.03
≥20%	-		-		**1.36**	**1.28-1.45**	**1.17**	**1.10-1.25**	**1.11**	**1.04-1.19**
	Neighborhood random effects									
Variance (se)	.5086 (.0160)		.4843 (.0155)		.4994 (.0157)		.4826 (.0154)		.4410 (.0170)	
Median odds ratio	1.97		1.94		1.96		1.94		1.88	

^a^Model 1 is an empty model.

^b^Model 5 includes the following covariates: sex, urban/rural, Medicaid, comorbidity, prior hospitalization, preventable hospitalization, endoscopies in prior year, stage, grade, histology, and tumor location.

Values in bold represent odds ratios for which the 95% confidence interval does not include the value one.

### Emergency surgery

Patients in the surgery sample (n = 55,046) were nested within 12,886 census tracts (range: 1–66 patients per tract). In all unadjusted and adjusted main effects models, African Americans, compared to whites, and those living in the highest poverty census tracts, compared to those in the lowest poverty tracts, were more likely to have emergency surgery. In the adjusted main effects model (Model 5), African Americans (vs. whites OR: 1.33 [95% CI: 1.23-1.44]) and those living in the highest poverty census tracts (≥20% vs. <10% poverty: OR: 1.11 [95% CI: 1.04-1.19]) were more likely to have emergency surgery.

### Race by neighborhood poverty interactions for emergency diagnosis and surgery

Race by neighborhood poverty interactions (p < .001) were evident for both outcomes in the final adjusted interaction models (Model 6). Table 3 presents the number of patients and the percent with both outcomes by group as well as the adjusted race and poverty interaction effects. In the interaction models, neighborhood poverty was no longer associated with either outcome among whites. African Americans living in the lowest poverty neighborhoods were equally likely to have emergency outcomes as whites living in similar conditions. Compared to whites in the lowest poverty neighborhoods (<10%), African Americans living in neighborhoods with ≥10% poverty rate demonstrated higher odds of both outcomes. African Americans living in the highest poverty neighborhoods (≥20%) had the greatest odds of both emergency diagnosis (AOR: 1.50, 95% CI: 1.38-1.63) and surgery (AOR: 1.63, 95% CI: 1.47-1.81).

**Table 3:** **Interaction between race and neighborhood poverty rate on emergency diagnosis and emergency surgery and neighborhood random effects in adjusted race and poverty interaction models (Model 6)** ^**a**^

	Emergency diagnosis				Emergency surgery			
	Total	Percent emergencies	Odds ratios and 95% confidence intervals		Total	Percent emergencies	Odds ratios and 95% confidence intervals	
White								
<10%	48767	28.1	1		32922	25.0	1	
10-19.9%	18903	27.3	0.97	0.92-1.02	12433	24.0	0.99	0.93-1.05
≥20%	8170	29.9	1.04	0.98-1.12	5245	26.8	1.06	0.98-1.15
Black								
<10%	1430	33.9	1.09	0.95-1.25	837	31.1	1.09	0.92-1.30
10-19.9%	1800	35.8	**1.16**	**1.02-1.31**	1111	34.6	**1.27**	**1.09-1.47**
≥20%	4260	42.4	**1.50**	**1.38-1.63**	2498	40.8	**1.63**	**1.47-1.81**
Variance (se)			.4881 (.0157)				.6254 (.0221)	
Median odds ratio			1.95				2.13	

^a^Models adjusted for the following covariates: sex, urban/rural, Medicaid, comorbidity, prior hospitalization, preventable hospitalization, endoscopies in prior year, stage, grade, histology, and tumor location, and the main effects of race and neighborhood poverty.

Values in bold represent odds ratios for which the 95% confidence interval does not include the value one.

### Neighborhood variation

Statistically significant variability in emergency diagnosis was present across census tracts for both outcomes in all models (Tables 2 and 3). In empty models (including no predictor variables), the MOR for census-tract variability in emergency diagnosis was 1.87. This can be interpreted as follows: if a patient switched from living in a randomly selected tract with low emergency diagnosis rate to randomly selected tract with a higher emergency diagnosis rate, her odds of emergency diagnosis would be 1.87 times higher (in the median). Neighborhood variability in emergency surgery (MOR = 1.97) was of similar magnitude. Neighborhood variability persisted, with similar magnitude, in multivariable models, suggesting that none of the covariates, including census tract poverty rate, accounted for census-tract variability in either outcome.

In sensitivity analyses, we examined, separately, two different indicators of both emergency diagnosis and surgery (data not shown). For both outcomes, in all models, African Americans and those living in higher poverty neighborhoods had higher odds of both 1) obstruction and/or perforations; and 2) emergency admissions (p < .001 for all comparisons).

## Discussion

To our knowledge this is the first U.S. population-based study to demonstrate neighborhood variation and race and SES disparities in both emergency CRC diagnosis and surgery. Observed disparities persisted after adjustment for a diverse array of socioeconomic, clinical, and tumor covariates. Among the strengths of this study is that we utilized multilevel models to demonstrate neighborhood variation in emergency CRC diagnosis and surgery.

Observed disparities and neighborhood variation in CRC emergencies indicate that breakdowns are occurring across the CRC diagnosis and treatment continuum that are systematically influenced by race, poverty, and/or neighborhood. Conceptual models of the CRC diagnosis and treatment continuum provide a framework for understanding *where* these break-downs are occurring. For example, the CRC screening process continuum delineates *transitions* from risk assessment to screening, detection, diagnosis, and treatment for asymptomatic patients undergoing CRC screening [39]. Likewise, for symptomatic CRC patients, The Aarhus statement [40] reviews models [41, 42] that describe the events and time intervals leading from CRC symptom appraisal and detection to diagnosis and treatment. There may be multiple identifiable break-down points across these continuums contributing to suboptimal screening uptake or delayed diagnosis and/or treatment, thus leading to CRC emergencies. Future interventions designed to prevent CRC emergencies and eliminate disparities will need to explicitly acknowledge the role of race, SES, and neighborhood influences throughout the CRC continuum.

Race and SES disparities evident in our study have been observed in some but not all previous U.S. studies, and no studies to our knowledge have tested whether neighborhood poverty affects emergency outcomes in African Americans and whites equally. Previous large, multi-state U.S. studies have demonstrated that African Americans face higher risk of emergency resection [6, 7]. In one of these same studies, however, zip code median income was not associated with emergency resection [6]. In another study from Michigan, African American race and lower census tract median annual household income were associated with increased odds of emergency CRC diagnosis in bivariate but not in multivariate analyses [21]. Differences in study design, populations, and case ascertainment may explain these divergent findings. For example, the Michigan study did not measure the urgency/emergency of the CRC diagnosis itself, but classified patients as to whether they had an ED visit within the month of or month before CRC diagnosis [21]. Further, other studies used zip code- or census tract- level median income, whereas we used a measure of census tract poverty rate with a priori cut-points that allow for nonlinear trends, as recommended by Krieger et al. [27, 43, 44].

There are several possible explanations for the interactive effects of race and neighborhood poverty we observed. African American and disadvantaged, low-SES populations are less likely to have obtained and maintained cancer screening than their counterparts [10–12]. They also may delay seeking care or experience health system delays once engaged in the healthcare system. These populations are less likely to have a regular primary care physician and have reduced access to care [45–47]. However, emerging evidence suggests that insurance and access to care alone do not entirely explain observed disparities [48, 49]. Even in a study of Medicare beneficiaries with a usual physician, rates of recent CRC screening were considerably lower among patients with low SES [49, 50]. Other factors such as patient-physician communication, discrimination in health care, logistical challenges such as transportation, the ability to take time off of work, and capacity to navigate health system bureaucracies may also play a role. The race by poverty interaction (p < .001) whereby poverty rate was associated with both outcomes only among African Americans suggests that African Americans in high poverty neighborhoods face additional, substantial barriers to CRC screening and/or timely diagnosis. More research is needed to better understand the dual effects and mechanisms of neighborhood poverty and race on CRC outcomes.

Notably, the neighborhood variability observed in our study persisted even after controlling for all covariates, including neighborhood poverty rate, indicating significant unexplained neighborhood variation in both outcomes. These results confirm a growing body of research suggesting that CRC outcomes, including screening, incidence, stage, and mortality, vary across geography [17, 19, 51–53]. For example, a prior study identified significant census-tract level variation in CRC survival that remained unexplained after accounting for individual covariates and neighborhood SES [19]. A more recent study identified census tract level variation in CRC screening that persisted even after accounting for the impact of physician and clinic- level influences using cross-classified statistical models [53]. Observed geographic variations in CRC outcomes remain poorly understood but may be a result of many factors such as practice patterns, managed care spillover, endoscopic capacity, or organizational culture within and across health systems.

Our finding of race and neighborhood SES disparities provides additional evidence of the disproportionate burden of CRC morbidity and mortality borne by African Americans and low-SES populations. CRC emergencies result in both short-and long-term negative impacts on morbidity, including postoperative complications, length of hospital stay, hospital readmissions, treatment cost, and mortality, [6, 7, 54–56] and likely also influence psychosocial outcomes and quality of life. The higher number of CRC emergencies among African Americans in high poverty neighborhoods may account for some of the disproportionate burden in CRC morbidity and mortality in these populations. Future research should examine interactions between race and poverty and should explore the extent to which race and SES disparities in emergency diagnosis account for observed disparities in morbidity and mortality.

It is difficult to directly compare the rate of emergencies we observed to other studies given different sampling strategies, populations, time periods under study, and methods of ascertaining CRC emergencies. Among 4 population-based U.S. studies, numbers range considerably. A National Inpatient Sample study reported 10.6% of CRC patients aged 65 and older had emergency resections in 2002, defined as surgery in the presence of perforation, peritonitis, or obstruction [6]. In Michigan, 23% of CRC patients aged 66 years and older, diagnosed 1996–2000, had emergency diagnoses [21]. Using a measure of inpatient resection acuity, a SEER-Medicare study of Stage I-III colon cancer patients diagnosed 1996–2003 found that 40% of patients had urgent/emergent resections [7]. Another SEER-Medicare study of colon cancer patients with adenocarcinoma diagnosed 1991–1996, found that 19.7% had emergent hospitalizations and 9.1% had obstruction or perforation [26]. This last study by Schrag et al. used the same ICD9 codes as in the present study, together with the same or similar Medicare claim code for emergent hospital admission [26]. A validated and widely used measure of emergent diagnoses and surgeries would facilitate comparisons across populations and over time.

Emergency colorectal cancer resection has been identified as “the clearest evidence on an individual level for a failure of screening” [54]. Thus, CRC emergencies may be an indicator to monitor progress in cancer screening initiatives over time [4, 24, 57]. In the absence of CRC screening, CRC emergencies may be the consequence of delays occurring after symptom presentation [40]. Accordingly, monitoring CRC emergencies over time may also provide insight into factors influencing diagnostic or treatment delays, such as access to care. Notably, unlike a Canadian study that documented a 24% relative decrease (from 24-18%) in emergency CRC surgeries between 1993–2001, [24] we found no significant change in either emergency outcome over time (p > .05). Given that an average of 10–15 years elapse between adenomatous polyp development and invasive cancer, [58, 59] and that Medicare began covering some CRC screening modalities in 1998, adding colonoscopy for screening of average risk adults in 2001, [60] it may be too soon to detect potential downward trends in CRC emergencies. Given recent shifts in economic and health policy trends, it is unclear whether race and SES disparities in CRC emergencies will persist, diminish, or potentially increase over time.

Our results should be interpreted in light of several limitations. First, we include only Medicare-insured patients aged 66 or older and therefore cannot generalize to younger or uninsured patients. Second, although we searched claims for symptoms and codes indicating emergencies in previous studies, [6, 7, 9, 24–26] some misclassification may have occurred. For example, we may have missed additional emergencies indicated by rectal bleeding (the severity of which cannot be measured with Medicare claims), in which case true incidence may be higher. Last, while we measured the number of lower endoscopies in the pre-diagnosis year, we could not measure complete CRC screening history nor endoscopic procedure quality, nor could we distinguish between screening or diagnostic procedures.

Despite these limitations, our study has several advantages over previous research. We conducted a multilevel study using a large population-based sample of U.S. CRC patients, used complete inpatient and outpatient claim data, and were able to adjust for a diverse array of tumor, patient, and neighborhood-level covariates. We further provided the first evidence of neighborhood variation and race and SES disparities in both emergency diagnosis and surgery.

## Conclusions

We documented neighborhood, race and SES disparities in CRC emergencies, where African Americans living in high poverty neighborhoods have the highest odds of two emergency outcomes and where significant neighborhood variation remains unexplained. Targeted interventions to increase screening in these vulnerable populations would reduce these preventable disparities.

## Abbreviations

CRC: Colorectal cancer screening
SES: Socioeconomic status
OR: Odds ratio
AOR: Adjusted odds ratio
SEER: Surveillance epidemiology and end results program.
